# Cross-sport patterns of health-related conditions in Chinese young athletes based on a comprehensive motor function assessment

**DOI:** 10.3389/fpubh.2026.1810653

**Published:** 2026-04-02

**Authors:** Zelong Miao, Beibei Wei, Congwen Cui, Hanping Zhang, Xuan Wang, Chaohui Xing, Liang Geng, Yuxiang Tang, Tianxiang Wang, Yi Liu, Bin Wang, Bin Jia

**Affiliations:** 1 School of Teacher’s Education, Hainan Normal University, Haikou, China; 2 Beijing DCN Orthopaedic Hospital, Beijing, China; 3 Department of Computer Science, Macao Polytechnic University, Macao, Macao SAR, China; 4 MOE Key Laboratory of Bioinformatics, Center for Synthetic and Systematic Biology, School of Life Sciences, Tsinghua University, Beijing, China

**Keywords:** athletic performance, injury prevention strategies, motor function assessment, sport-specific risk, youth sports injuries

## Abstract

**Background:**

The increasing global participation in youth sports has been accompanied by a concerning rise in sports-related injuries among children and adolescents, highlighting the need for more comprehensive assessment approaches. Current methods often fail to account for the complex interplay of biomechanical, physiological, and developmental factors unique to young athletes.

**Methods:**

This study employed an integrated motor function assessment framework combining sport-specific risk evaluation, Functional Movement Screening (FMS), metabolic-nutritional profiling, and biomechanical analysis (plantar pressure/gait) to address this gap. We included 307 participants (213 males and 94 female young athletes) from primary and secondary schools in Haidian District, Beijing, China.

**Results:**

Analysis of 11 sports revealed distinct injury patterns: 78% of male injuries involved lower extremities (soccer 38% foot/ankle, basketball 32% knee), while females showed combined lower/upper body issues (volleyball 19% shoulder). Sex differences were most pronounced in combat sports, where males demonstrated approximately 2 times higher lower limb injury prevalence compared to females. These findings highlight the critical need for sex-specific prevention strategies, recommending targeted interventions for male athletes’ kinetic chain stability and female athletes’ proprioceptive-neuromuscular control. The study provides descriptive epidemiological data that may inform the development of sport-specific, sex-optimized training regimens.

**Conclusion:**

These findings suggest that the proposed integrated assessment framework may offer a useful approach for identifying sport- and sex-specific injury patterns in youth athletes. The observed associations between motor function impairments and injury distribution provide preliminary evidence to inform the development of targeted prevention strategies. Further prospective studies are warranted to evaluate the effectiveness of this framework in reducing injury risk and supporting long-term athletic development.

## 1 Introduction

The global emphasis on health and fitness has led to a surge in sports participation, both competitively and recreationally. However, this rise in engagement has been accompanied by an increase in sports-related injuries, creating significant challenges for athletes, healthcare systems, and sports organizations. Annual statistics indicate that millions of athletes are forced to suspend or abandon training and competition due to such injuries, with profound consequences for individual careers as well as socioeconomic burdens on teams and medical institutions (1).

Research highlights distinct injury patterns across sports. Organized contact sports (e.g., soccer, basketball, soccer) exhibit higher injury rates, primarily sprains, strains, and fractures, compared to unstructured activities (2). The male adolescents face elevated risks in certain sports, likely due to participation intensity and biomechanical factors (3). Sport-specific variations are evident: team sports like soccer show higher competition-phase injuries, with sex disparities—females are prone to ACL tears and ankle sprains, while males experience more muscle strains and contact injuries (4, 5). Tennis players often suffer overuse injuries (e.g., rotator cuff tendinitis) (6), whereas weight-training athletes frequently encounter acute strains and chronic tendinitis (7). Combat sports like judo mirror rugby’s injury rates (8), and winter sports such as snowboarding carry high risks of fractures and dislocations (9). Key risk factors include biomechanical deficiencies, physiological differences, and training errors, necessitating tailored preventive strategies like neuromuscular training and technique optimization (10, 11).

Motor function assessment is critical for injury prevention and performance optimization. Established tools like the Bruininks-Oseretsky Test (BOT-2) and Functional Movement Screening (FMS) evaluate coordination, stability, and injury risk, with applications in clinical and athletic settings (12, 13). Sex-specific differences emerge in movement quality; for instance, male athletes often exhibit greater functional asymmetries, while females demonstrate superior stability (14). Advanced methods, such as gait-cycle energy analysis (15) and plantar pressure mapping (16), further refine movement assessment. Foot biomechanics also play a pivotal role: flat or high-arched feet alter pressure distribution, influencing injury risks like plantar fasciitis or stress fractures (17)—a concern exacerbated in adolescents with developing musculoskeletal systems (18).

Despite these advancements, traditional assessments often focus on isolated metrics (e.g., strength, range of motion), lacking integration of multifactorial risk determinants. This study addresses this gap by presenting a multidimensional motor function assessment framework that synthesizes sports risk evaluation, FMS, metabolic-nutritional profiling, plantar pressure analysis, and gait biomechanics. Through descriptive analysis of cross-sectional data, our approach aims to identify sport-specific injury patterns that may inform future targeted interventions. By bridging these domains, this research seeks to enhance injury prevention strategies, optimize athletic performance, and improve long-term athlete wellbeing.

## 2 Methods

### 2.1 Study participants and design

A cross-sectional study was conducted to examine sport-related injury patterns across multiple youth athletic populations. A total of 307 participants (213 males and 94 female young athletes) from 11 sports—soccer, basketball, baseball, handball, tennis, volleyball, hockey, track and field, swimming, judo, and wushu taolu—underwent comprehensive motor function assessments.

In accordance with the IOC consensus statement (19), we defined injuries as conditions that received medical attention, regardless of whether they resulted in time loss from training or competition, and distinguished between medical-attention injuries, which are acute or overuse conditions diagnosed by clinical examination; structural abnormalities, such as anatomical variations like hallux valgus or flat feet identified during physical examination, which are considered potential risk factors rather than injuries unless accompanied by pain or functional impairment; and functional impairments, including asymmetries or movement deficiencies identified during FMS or balance testing, classified as risk indicators rather than injuries.

From February 2025 to May 2025, Beijing DCN Orthopedic Hospital conducted a study involving external subjects recruited from schools in Haidian District, Beijing. Participants were required to meet the following inclusion criteria: aged 7–18 years (regardless of sex), enrolled in primary or middle school, capable of participating in team sports activities, proficient in Mandarin, and willing to voluntarily participate in the study.

All methods in this study were carried out in accordance with relevant guidelines and regulations. The experimental protocol was approved by the Ethics Committee of Beijing DCN Orthopedic Hospital (Approval no. JD2022-02). Written informed consent was obtained from all participants and their legal guardians after being fully informed about the study details.

The sample size was determined based on available resources and the exploratory nature of this descriptive study. No formal power analysis was conducted. We acknowledge that subgroup analyses by sport and sex are limited by small sample sizes, which restricts our ability to draw definitive statistical inferences. Therefore, this study should be considered exploratory, and findings should be interpreted with caution.

### 2.2 Athletic risk assessment

We implemented a comprehensive risk assessment protocol to systematically evaluate athletes’ health status: The process began with training history analysis, examining athletes’ training programs, injury records (including injury types, frequency, and recovery status), and training load quantification. Subsequently, certified sports medicine physicians conducted standardized functional musculoskeletal examinations following established protocols, assessing key indicators including bone integrity, joint stability, ligament laxity, and muscular balance in the shoulders, cervical spine, thoracolumbar spine, elbows, wrists/hands, hips, knees, and ankles.

### 2.3 Athletic performance assessment

The FMS protocol (20) was employed to assess movement quality through seven standardized tests: Deep Squat (evaluating full-body mobility/symmetry), Hurdle Step (testing hip mobility/single-leg stability), In-Line Lunge (measuring trilateral joint stability), Shoulder Mobility (assessing scapulothoracic-glenohumeral coordination), Active Straight-Leg Raise (examining hamstring flexibility/core stability), Trunk Stability Push-Up (evaluating core strength/spinal stabilization), and Rotary Stability Test (analyzing multi-segmental coordination), with each test scored 0–3 (3 = perfect execution, 2 = compensated movement, 1 = task failure, 0 = pain occurrence) and composite scores below 14/21 indicating elevated injury risk (21) requiring corrective interventions, while neuromuscular control was evaluated through three complementary assessments: the Y-Balance Test (YBT) measuring dynamic balance via maximal reach distances in anterior, posteromedial, and posterolateral directions (with limb asymmetry >4 cm or composite scores <94% suggesting increased injury risk) (22), high-speed video analysis (240fps) of change-of-direction drills to examine foot strike angle, knee valgus during deceleration, and trunk control, and proprioceptive testing through single-leg stance (eyes closed) on force plates where postural sway exceeding 5.2cm^2^ (95% confidence ellipse area) indicated deficient joint position sense (23).

All assessments were conducted by trained and certified professionals. The athletic risk assessments and functional musculoskeletal examinations were performed by two licensed sports medicine physicians with over 5 years of clinical experience. The FMS, Y-Balance, and biomechanical assessments were conducted by three certified athletic trainers or physiotherapists. These assessors were blinded to the athletes’ injury history and to the results of other assessments (e.g., the physicians were unaware of the FMS scores, and the trainers were unaware of the clinical diagnosis) to minimize bias.

### 2.4 Metabolic and nutritional assessment

The metabolic and nutritional assessment was conducted using bioelectrical impedance analysis (X-Scan Plus II, JAWON, Korea) to measure core body composition parameters including fat mass percentage, visceral fat rating (1–15 scale), and skeletal muscle mass (kg), along with derived metrics such as phase angle (indicating cellular health) and extracellular-to-intracellular water ratio, with all measurements performed in accordance with ISAK guidelines requiring morning testing in a fasted state, at least 48 h post-exercise, and following standardized hydration protocols.

### 2.5 Plantar pressure assessment and gait analysis

This study utilized the FreeMed™ plantar pressure and posture assessment platform (Sensor Medica s.a.s., Italy), a 1.2 m × 0.8 m pressure distribution system with high-sensitivity sensors and a sampling frequency >400 Hz. The FreeMed™ platform was calibrated daily according to the manufacturer’s instructions using the integrated auto-calibration procedure prior to any data collection to ensure measurement accuracy and sensor sensitivity. For static assessment, participants stood naturally on the platform for 3–4 s. For dynamic gait analysis, participants walked naturally along a walkway for five round trips. The screening protocol was designed based on professional biomechanical theory and the target population’s age range, utilizing the Foot Posture Index-6 (FPI-6) as a foundational framework. The FPI-6 is a clinically validated tool for quantifying static foot alignment and postural abnormalities, with established reliability, validity, sensitivity, and specificity. Participants were assessed in the Relaxed Calcaneal Stance Position (RCSP). Three key observational parameters recorded: (1) the frontal-plane angle of the calcaneus relative to the tibia, (2) the visibility of lateral toes from a posterior view (indicative of subtalar pronation), and (3) forefoot morphology, including the presence of Morton’s Foot (characterized by a hypoplastic first metatarsal and elongated second/third metatarsals) or transverse arch collapse.

The combined findings of (1) and (2) were used to classify subtalar joint alignment as excessive pronation, excessive supination, or neutral (no significant deviation). This classification was further interpreted through a structured pain history interview, documenting the location, nature, duration, and triggers of lower-limb movement-related pain, along with aggravating or alleviating factors. Forefoot structural anomalies (e.g., Morton’s Foot or transverse arch collapse) were evaluated for their association with active or historical pain to identify potential injury mechanisms.

Participants were categorized as normal (neutral subtalar alignment without movement-related pain), as having a structural variation only (subtalar deviation or forefoot anomalies without current pain), or as having an injury (subtalar deviation or forefoot anomalies accompanied by current pain, or any medically-attended condition regardless of structural findings).

### 2.6 Data integration and statistical analysis

The assessment integrates findings from movement patterns, dynamic stability, and neuromuscular control tests to identify asymmetries (e.g., strength or mobility imbalances between sides) and compensatory movements that could contribute to overuse injuries. It is important to emphasize that this integration is descriptive rather than predictive. We present associations between assessment findings and injury patterns without claiming causal relationships or validated risk prediction.

Statistical analyses were performed using SPSS Statistics version 27.0 and graphing was conducted using GraphPad Prism software, version 9.0 with a two-tailed *α* level of 0.05 set as the threshold for statistical significance and exact *p*-values reported where applicable to accommodate sparse data structures. Given the exploratory nature of this study and the small subgroup sample sizes, we did not adjust for multiple comparisons, acknowledging that this may increase the risk of Type I error. Findings should therefore be interpreted with appropriate caution. Descriptive statistics were calculated for demographic and clinical variables, with prevalence rates presented as percentages. For the youth male soccer, basketball, baseball, handball cohort, a Chi-square goodness-of-fit test was conducted to determine whether the distribution of injury cases across anatomical sites deviated from a uniform distribution, with standardized residuals computed to identify sites contributing most to significant deviations and exact *p*-values reported due to expected cell frequencies below 5. To examine sex differences in injury prevalence, a Cochran–Mantel–Haenszel (CMH) test was employed for each sport, wherein data were structured as a series of 2 × 2 contingency tables (sex × injury status) stratified by body part; the Mantel–Haenszel common odds ratio (OR_MH) and its 95% confidence interval were calculated to estimate the overall sex effect across sites, and the Breslow–Day test was used to assess homogeneity of odds ratios across strata, with a non-significant result (*p* > 0.05) supporting the validity of the pooled estimate. Site-specific sex comparisons were conducted descriptively using Fisher’s exact test due to low expected frequencies in several strata.

## 3 Results

### 3.1 Overall injury distribution characteristics

The analysis revealed consistent lower limb predominance in sports health-related conditions, with the foot (10/11 teams), knee (9/11), and ankle (8/11) being most affected, reflecting their substantial biomechanical load. Distinct sport-specific patterns emerged: ball sports showed stress-related lower extremity injuries; racket/throwing sports demonstrated upper body involvement (shoulders, wrists, core) from repetitive arm motions; while combat sports exhibited foot-knee complex injuries due to impact forces. These findings highlight how injury distribution directly correlates with sport-specific biomechanical demands, underscoring the need for tailored prevention programs focusing on joint stability, neuromuscular control, and functional strengthening of high-risk areas specific to each sport’s movement patterns (Table 1).

**Table 1:** Distribution of health-related conditions by sport and sex.

Sport	Sex	*N*	Knee	Ankle	Foot	Shoulder	Spinal/Lumbar	Hip/Pelvis	Wrist/Hand	Lower limb alignment	Other muscular/functional
Soc	M	39	2	6	9	1	1	3	1	5	0
	F	0	–	–	–	–	–	–	–	–	–
Bas	M	22	7	4	1	1	1	0	1	0	0
	F	0	–	–	–	–	–	–	–	–	–
Bsb	M	18	0	0	8	0	0	0	0	2	12
	F	0	–	–	–	–	–	–	–	–	–
Han	M	30	2	4	0	1	1	0	1	0	3
	F	0	–	–	–	–	–	–	–	–	–
Ten	M	10	4	1	2	1	0	0	1	3	0
	F	6	1	0	1	0	0	0	0	0	0
Vb	M	11	2	4	1	0	0	0	0	4	0
	F	26	7	2	1	5	3	0	1	5	0
Hoc	M	18	3	1	11	0	0	0	0	0	0
	F	16	0	2	9	1	1	0	0	0	2
T&F	M	14	0	2	1	0	0	0	0	0	2
	F	11	0	0	0	0	1	0	0	0	0
Swim	M	17	1	0	12	0	1	0	0	0	0
	F	16	0	0	9	1	0	0	0	1	1
Jud	M	28	9	5	18	0	1	0	0	4	2
	F	14	0	1	4	0	1	0	0	0	2
WT	M	6	3	2	3	0	0	0	0	0	0
	F	5	2	0	2	3	2	1	1	0	0

Data are presented by sport and sex (M: Male; F: Female) with the number of health-related conditions (N) per anatomical site. Injury sites include: Knee, Ankle, Foot, Shoulder, Spinal/Lumbar, Hip/Pelvis, Wrist/Hand, Lower Limb Alignment, and Other Muscular/Functional. Sport abbreviations: Soc (Soccer), Bas (Basketball), Bsb (Baseball), Han (Handball), Ten (Tennis), Vb (Volleyball), Hoc (Hockey), T&F (Track and Field), Swim (Swimming), Jud (Judo), WT (Wushu Taolu).

### 3.2 Discipline-specific injury pattern

**Figure 1:**
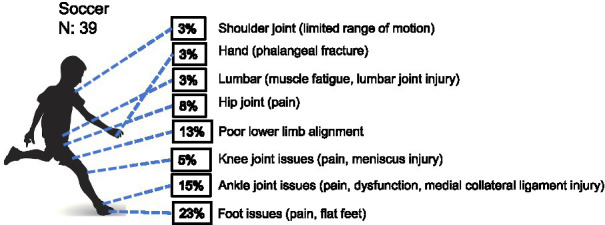
Prevalence of health-related conditions in soccer players (*N* = 39). Data are presented as percentages of players reporting each condition. The same below.

*Soccer*: of the 39 youth male soccer players assessed, 11 were found to be injury-free with normal examination results. The remaining 28 cases of sports-related health-related conditions were analyzed using a chi-square goodness-of-fit test. The results revealed a significantly uneven distribution of injury frequencies across different body regions (*χ*^2^ = 17.143, df = 7, *p* = 0.016), indicating a high degree of site specificity in injury patterns within this population. Specifically, the foot accounted for the highest proportion of health-related conditions (*n =* 9), with an observed frequency significantly exceeding the expected count under the assumption of uniform distribution (3.5 cases), and a standardized residual of +2.9—marking it as the primary contributor to the overall statistical difference. Ankle injuries (*n =* 6) and lower limb injuries (*n =* 5) also showed notably elevated trends. In contrast, injuries to the shoulder, hand, and lumbar region each occurred in only one case, all falling significantly below expected levels (Table 1 and Figure 1). These findings indicate that sports-related injuries in youth male soccer players are highly concentrated in the distal lower extremities, with the foot and ankle being the most prominent sites.

**Figure 2:**
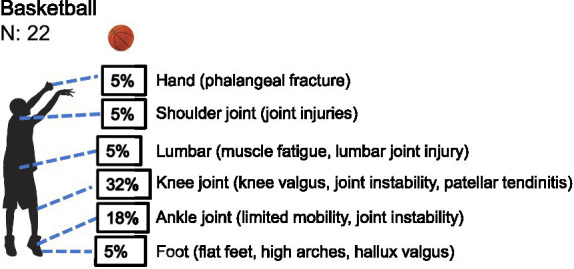
Prevalence of health-related conditions in basketball players (*N* = 22).

*Basketball*: among the 22 youth male basketball players assessed, the distribution of health-related conditions across different body regions was found to be significantly uneven (*χ*^2^ = 12.600, df = 5, *p* = 0.027). Specifically, knee injuries accounted for the highest proportion (*n =* 7), with an observed frequency substantially exceeding the expected count under the assumption of uniform distribution (2.5 cases) and a standardized residual of +4.5—identifying the knee as the primary contributor to the overall statistical difference. Ankle injuries (*n =* 4) also exhibited a notably elevated trend (standardized residual = +1.5). In contrast, injuries to the shoulder, foot, hand, and lumbar region each occurred in only one case, all falling below expected levels (Table 1 and Figure 2). These findings indicate that health-related conditions in youth male basketball players are highly concentrated in the lower extremities, with the knee being the most prominent site, followed by the ankle .

**Figure 3:**
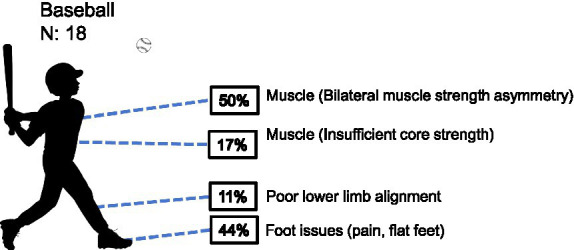
Prevalence of health-related conditions in baseball players (*N* = 18).

*Baseball*: among the 18 youth male baseball players assessed, the overall distribution of injury types did not reach statistical significance (*χ*^2^ = 6.727, df = 3, *p* = 0.081). Examination of specific injury categories revealed that bilateral muscle strength asymmetry (*n =* 9) and foot abnormalities (*n =* 8) were the two most prevalent conditions, with observed frequencies exceeding the expected count under the assumption of uniform distribution (5.5 cases), and standardized residuals of +3.5 and +2.5, respectively. In contrast, limb injuries (*n =* 2) and insufficient core strength (*n =* 3) occurred at frequencies below expected levels. Although the omnibus test did not achieve statistical significance, the marked concentration of bilateral strength asymmetry and foot abnormalities suggests notable clinical trends (Table 1 and Figure 3). These findings indicate that the injury profile of youth male baseball players is characterized by a combination of functional imbalance and distal lower extremity load.

**Figure 4:**
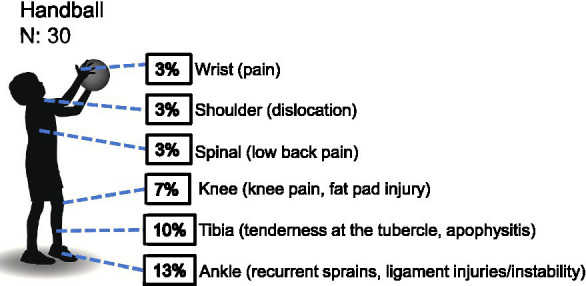
Prevalence of health-related conditions in handball players (*N* = 30).

*Handball*: among the 30 youth male handball players assessed, no statistically significant difference was observed in the distribution of injury frequencies across different body regions (*χ*^2^ = 4.000, df = 5, *p* = 0.549). Specifically, the ankle accounted for the highest proportion of injuries (4 cases), followed by the tibia (3 cases) and the knee (2 cases). Injuries to the shoulder, spine, and wrist each occurred in one case. Although the observed frequencies for the ankle and tibia were slightly higher than the expected count (2.0 cases), the knee matched the expected value, and the remaining regions fell slightly below, none of these deviations reached statistical significance (Table 1 and Figure 4). Based on these descriptive findings, injury prevention efforts in youth male handball might consider prioritizing ankle stability training, landing posture control, and proprioceptive exercises, while also addressing the risk of tibial stress injuries.

**Figure 5:**
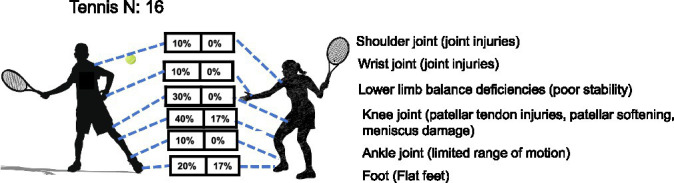
Prevalence of health-related conditions in tennis players (*N* = 16).

*Tennis*: a total of 16 youth handball players (10 males, 6 females) were assessed for health-related conditions across body regions. Due to the small sample size and the presence of multiple injuries per individual, Fisher’s exact tests were conducted to examine sex differences in injury prevalence for each site. Results indicated that although male players exhibited higher injury rates in all regions—most notably the knee (40% vs. 16.7%) and foot (20% vs. 16.7%)—none of these differences reached statistical significance (all *p* > 0.05, Fisher’s exact test). The lack of significance is likely attributable to the limited sample size, particularly the small number of female athletes (Table 1 and Figure 5). These findings suggest that while sex-specific injury patterns may exist, larger-scale studies are needed to confirm whether the observed disparities reflect true biological or biomechanical differences. Currently, injury prevention programs for youth tennis players should continue to emphasize lower extremity injury prevention, particularly for the knee and foot, in both male and female athletes.

**Figure 6:**
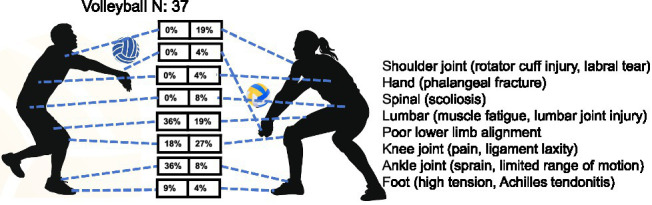
Prevalence of health-related conditions in volleyball players (*N* = 37).

*Volleyball*: a total of 37 youth volleyball players (11 males, 26 females) were assessed for health-related conditions. The results revealed a statistically significant overall sex difference in injury distribution (Mantel–Haenszel *χ*^2^ = 6.82, df = 1, *p* = 0.009), with a common odds ratio of 0.41 (95% CI: 0.21–0.80), indicating that female athletes had significantly higher odds of injury across sites compared to males. The Breslow–Day test was not significant (*p* = 0.27), suggesting that the sex effect was consistent across body regions. Region-specific analysis showed distinct injury patterns between sexes. Male athletes demonstrated lower limb-dominant injuries with high prevalence of ankle problems (36%) and lower limb malalignment (36%), followed by knee issues (18%). Female players displayed more complex injury patterns featuring predominant knee disorders (27%) and shoulder mobility limitations (19%), along with spinal (4%), hand (4%) and lumbar problems (8%) completely absent in males (0%). Notably, despite the female sample size (26) being 2.4 times larger than males (11), the overall incidence of lower limb injuries remained higher in male athletes (Table 1 and Figure 6). These findings indicate that sex-specific injury patterns exist in youth volleyball, with females exhibiting a broader injury profile involving both the upper and lower extremities and the axial skeleton, while males show a more concentrated burden in the lower extremities.

**Figure 7:**
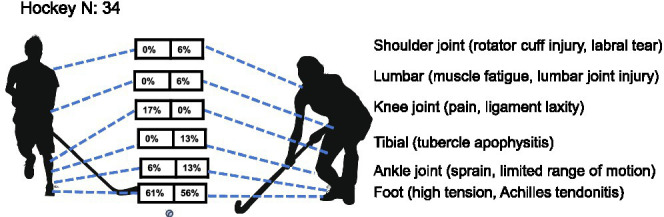
Prevalence of health-related conditions in hockey players (*N* = 34).

*Hockey*: a total of 34 youth field hockey players (18 males, 16 females) were assessed for health-related conditions. A Cochran–Mantel–Haenszel test stratified by body part revealed no statistically significant overall sex difference in injury distribution (MH *χ*^2^ = 0.173, df = 1, *p* = 0.677), with a common odds ratio of 1.22 (95% CI not calculated due to non-significance), indicating that the odds of injury did not differ significantly between female and male athletes. Male athletes predominantly exhibited foot disorders (61% hallux valgus/heel pain) and knee problems (17% valgus/pain), while female players also primarily experience foot issues (56%) but displayed a broader injury spectrum–including ankle (13%), shoulder (6%), lumbar fatigue (6%) and tibial problems (13%) completely absent in males (0%). Particularly noteworthy is the complete absence of knee problems in female samples (0%) contrasting with the 17% incidence in males (Table 1 and Figure 7).

**Figure 8:**
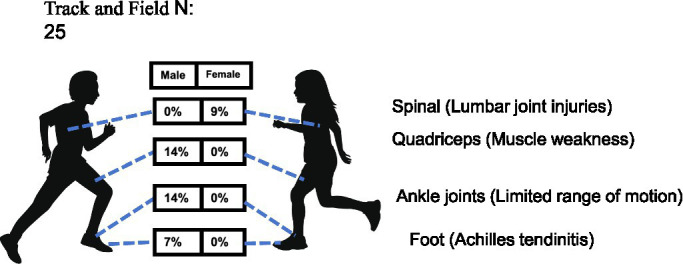
Prevalence of health-related conditions in track and field players (*N* = 25).

*Track and field*: a total of 25 youth track and field athletes (14 males, 11 females) were assessed for health-related conditions. The results revealed no statistically significant overall sex difference in injury distribution (MH *χ*^2^ = 1.871, df = 1, *p* = 0.171), with a common odds ratio of 0.25 (95% CI: 0.03–2.17), indicating that the odds of injury did not differ significantly between female and male athletes. Despite the lack of overall statistical significance, distinct injury patterns emerged between sexes. Notably, lumbar joint injuries were observed exclusively in female athletes, with an incidence rate of 9%. In contrast, male athletes demonstrated a higher susceptibility to musculoskeletal conditions, including quadriceps weakness (14%), limited ankle joint range of motion (14%), and Achilles tendinitis (7%)—none of which were reported in their female counterparts (Table 1 and Figure 8). These divergent injury profiles suggest the potential need for sex-tailored prevention and rehabilitation strategies in youth track and field, though these observations require confirmation in larger studies.

**Figure 9:**
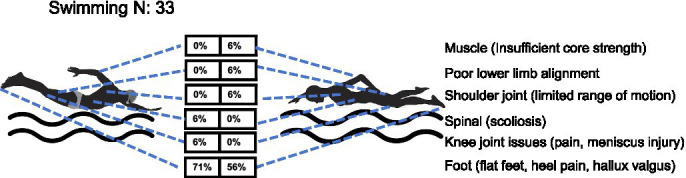
Prevalence of health-related conditions in swimming players (*N* = 33).

*Swimming*: a total of 33 youth swimming athletes (17 males, 16 females) were assessed for health-related conditions across six anatomical regions. The results revealed no statistically significant overall sex difference in injury distribution (MH *χ*^2^ = 0.115, df = 1, *p* = 0.735), with a common odds ratio of 0.83 (95% CI: 0.36–1.89), indicating that the odds of injury did not differ significantly between female and male athletes. Despite the lack of overall significance, distinct injury patterns were observed. Male athletes predominantly exhibited foot problems (71% hallux valgus/heel pain) and upper cross syndrome (47%), while female athletes, though also primarily affected by foot disorders (56%), demonstrated a broader injury profile including shoulder mobility limitations (6%), lower limb malalignment (6%), and core muscle weakness (6%) - all absent in males (0%). Notably, spinal issues (6%) and knee problems (6%) were exclusively observed in male swimmers, with females maintaining 0% incidence in these categories (Table 1 and Figure 9). These findings suggest that although sex-specific injury patterns may exist in youth swimming, the differences observed in this sample did not reach statistical significance, likely due to the limited sample size and the predominance of foot abnormalities in both groups.

**Figure 10:**
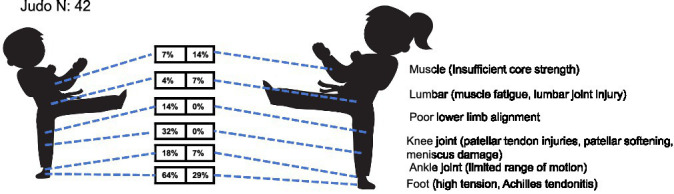
Prevalence of health-related conditions in judo players (*N* = 42).

*Judo*: a total of 42 youth judo athletes (28 males, 14 females) were assessed for health-related conditions. The results revealed a statistically significant overall sex difference in injury distribution (MH *χ*^2^ = 8.112, df = 1, *p* = 0.004), with a common odds ratio of 0.306 (95% CI: 0.135–0.694), indicating that female athletes had significantly lower odds of injury across sites compared to their male counterparts. Male athletes most frequently presented with foot disorders (64%, including hallux valgus and heel pain), followed by knee problems (32%, e.g., valgus deformity and pain), and flexibility-balance impairments (25%). In contrast, female athletes primarily exhibited flexibility-balance impairments (57%) and bilateral muscle imbalance (14%). Notably, lower limb malalignment (14%) and lumbar injuries (4%) were observed only in males, whereas females showed a slightly higher rate of lumbar issues (7%) but no cases of malalignment. Ankle (7%) and foot problems (29%) were considerably less common in females than in males (Table 1 and Figure 10).

**Figure 11:**
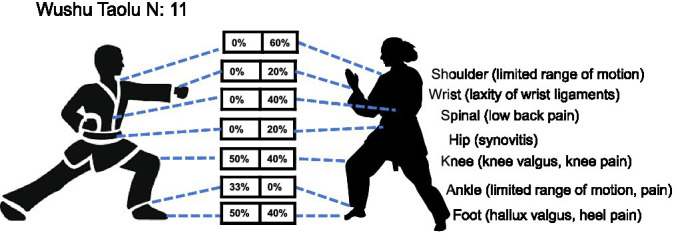
Prevalence of health-related conditions in Wushu Taolu players (*N* = 11).

*Wushu Taolu*: a total of 11 youth Wushu Taolu athletes (6 males, 5 females) were assessed for health-related conditions. The results revealed no statistically significant overall sex difference in injury distribution (MH *χ*^2^ = 1.610, df = 1, *p* = 0.204), with a common odds ratio of 1.93 (95% CI: 0.70–5.33), indicating that the odds of injury did not differ significantly between female and male athletes. Despite the lack of statistical significance due to the small sample, distinct injury patterns were observed. Males predominantly showed knee disorders (50%), and foot abnormalities (50%), while females exhibited higher rates of limited shoulder mobility (60%), low back pain (40%), and foot issues (40%). Notably, knee valgus/pain in males and shoulder problems in females were particularly prominent, with ankle problems only found in males (33%), and wrist, spinal and hip synovitis exclusively found in females (20, 40, and 20%, respectively) (Table 1 and Figure 11). These findings suggest that while sex-specific injury patterns may exist in youth Wushu Taolu, with males showing greater lower extremity involvement and females exhibiting a broader upper/lower extremity and spinal distribution, the differences observed in this sample did not reach statistical significance, likely attributable to the very limited sample size.

### 3.3 Sex differences in injury patterns

Analysis of injury patterns reveals consistent sex-based variations across multiple sports disciplines. Male athletes predominantly exhibit lower limb pathologies, showing high rates of foot disorders (soccer 23%, hockey 61%, judo 64%), knee problems (basketball 32%, tennis 40%), and ankle issues (volleyball 36%). Their injury profiles reflect concentrated biomechanical stress on weight-bearing joints, with functional chain disruptions (baseball bilateral imbalance 50%) being particularly notable. Conversely, female athletes demonstrate more diversified injury patterns combining lower extremity (volleyball knee 27%) with upper body involvement (volleyball shoulder 19%, Wushu Taolu 60%), along with higher incidence of postural control impairments (judo flexibility-balance 57%). These differential patterns may stem from anatomical variations, hormonal influences on connective tissue, and sport-specific movement mechanics, though causal mechanisms cannot be determined from this cross-sectional study. These observations underscore the potential value of sex-adapted prevention programs targeting male athletes’ lower limb stability and female athletes’ multi-joint neuromuscular control, though such strategies require prospective evaluation.

## 4 Discussion

### 4.1 Injury mechanisms and sports biomechanics

The high prevalence of lower limb injuries observed across multiple sports disciplines aligns with existing literature, emphasizing the biomechanical stresses imposed by dynamic movements such as jumping, abrupt stops, and rapid directional changes (24). These actions generate excessive shear forces, ligament overstretching, and muscle fatigue, potentially increasing susceptibility to acute and overuse injuries. Notably, even low-impact sports like swimming demonstrated chronic foot pathologies (e.g., flat feet) due to repetitive kicking motions, underscoring the potential need for sport-specific preventive strategies that address cumulative microtrauma.

### 4.2 Sport-specific risk factors

*Contact Sports* (Soccer/Basketball/Hockey): The high prevalence of lower limb injuries (soccer 38% foot/ankle, basketball 32% knee, hockey 61% foot disorders) may be associated with repetitive cutting, jumping, and collisions that overload passive stabilizers. The elevated ankle injury risk in these sports correlates with frequent collisions and pivoting maneuvers, which destabilize joints and strain passive stabilizers (ligaments). Our findings support Robles-Palazón et al. (3), who identified similar patterns in youth athletes, with males more prone to muscle strains and ankle sprains.

*Technique-Driven Sports* (Volleyball/Wushu Taolu/Swimming): Asymmetrical loading patterns in overhead motions (volleyball spikes, Wushu rotations) led to sex-divergent risks—females developed more shoulder (19%) and core instability injuries (6%), while males exhibited foot/ankle overuse (71% in swimming), reinforcing the potential value of sex-tailored interventions (3).

*Combat Sports* (Judo): The 114% higher lower limb injury risk in males versus females’ 71% neuromuscular impairments reflects sport-specific demands: explosive takedowns (male-dominant) versus sustained grappling (female-dominant), necessitating distinct prevention frameworks.

### 4.3 Prevention and intervention recommendations

Based on the descriptive patterns observed, the following intervention strategies may warrant consideration and prospective evaluation.

*Targeted training*: balance training (e.g., single-leg hops, proprioceptive drills) significantly reduces lower limb injury risks by enhancing neuromuscular control (24). For sports like basketball, incorporating quadriceps and core strengthening can optimize load distribution during landings.

*Movement optimization*: standardizing techniques (e.g., soft-landing postures in volleyball) minimizes aberrant joint stresses. Swimmers benefit from foot intrinsic muscle exercises (e.g., towel scrunches) to counteract arch collapse.

*Sex-specific strategies*: females require ACL injury prevention programs emphasizing neuromuscular control, while males need flexibility training and collision-protection drills (3). These recommendations are based on the observed patterns in our study and established literature, but their effectiveness requires confirmation through rigorous intervention studies.

### 4.4 Study limitations and future directions

This study has several important limitations that must be considered when interpreting. First, this is a cross-sectional study, which means we cannot establish causal relationships between motor function impairments and injuries. The associations we report should be interpreted as descriptive rather than predictive, and we cannot determine whether functional deficits preceded or resulted from injuries. Additionally, sample sizes are limited, particularly for sport-specific and sex-specific subgroups. This restricts statistical power and may have prevented detection of meaningful differences (Type II error). Estimates from small subgroups (e.g., Wushu Taolu, *n =* 11) are unstable and should be interpreted with caution. This study should be considered exploratory, and findings require validation in larger, adequately powered cohorts. Furthermore, we lack exposure data and did not calculate exposure-adjusted injury rates (e.g., injuries per 1,000 training/competition hours), which is the gold standard in sports injury epidemiology, limiting the precision and comparability of our prevalence estimates. Our injury definition, while aligned with IOC guidelines, also has limitations; despite distinguishing between injuries, structural variations, and functional impairments, retrospective reporting and medical record completeness may have introduced information bias. Moreover, we did not classify injury severity (e.g., mild, moderate, severe, or time-loss), limiting comprehensive understanding of injury impact, nor did we adjust for multiple comparisons, acknowledging that this increases the risk of Type I error. Results with *p*-values near 0.05 should therefore be interpreted with particular caution. Finally, the study was conducted in a single geographic region (Haidian District, Beijing, China), which may limit generalizability to other populations, cultural contexts, or training systems, and our multidimensional assessment framework is descriptive rather than predictive—we have not developed or validated a composite risk score or integration algorithm, and the framework should be viewed as a comprehensive descriptive tool rather than a validated predictive model.

Future research directions should address these limitations through prospective cohort designs that track athletes over time to establish temporal relationships and develop risk prediction models. Studies should also include exposure data to calculate accurate injury rates and employ larger, multi-center samples with adequate power for subgroup analyses. Integration of wearable technology to monitor real-time biomechanical loads, injury severity classification to assess clinical and functional impact, and formal validation of assessment frameworks as predictive tools are also needed. Finally, intervention studies testing whether targeted strategies based on assessment findings effectively reduce injury incidence will be essential to translate descriptive findings into practical prevention approaches.

## 5 Conclusion

This cross-sectional study describes the predominance of lower limb injuries across multiple youth sports, with distinct patterns associated with sport-specific biomechanics and sex. These descriptive findings suggest the potential value of further research into customized prevention strategies that account for sport-specific demands and sex-based physiological differences. However, given the exploratory nature and methodological limitations of this study, the findings should be interpreted with appropriate caution. The observed associations between motor function assessments and injury patterns provide preliminary descriptive evidence that may inform hypotheses for future prospective research. Implementing and rigorously evaluating targeted prevention approaches in well-designed intervention studies may ultimately contribute to improved injury risk management and long-term athlete wellbeing.

## Data Availability

The original contributions presented in the study are included in the article/Supplementary material, further inquiries can be directed to the corresponding author.
